# O’Connor et al. systematic review regarding animal feeding operations and public health: critical flaws may compromise conclusions

**DOI:** 10.1186/s13643-017-0575-7

**Published:** 2017-08-31

**Authors:** Keeve E. Nachman, Juleen Lam, Leah H. Schinasi, Tara C. Smith, Beth J. Feingold, Joan A. Casey

**Affiliations:** 1 Johns Hopkins Center for a Livable Future, Johns Hopkins University, Baltimore, MD USA; 2 Department of Environmental Health and Engineering, Bloomberg School of Public Health, Johns Hopkins University, 615 North Wolfe Street, Suite W7010-E, Baltimore, MD 21205 USA; 3 Johns Hopkins Risk Sciences and Public Policy Institute, Baltimore, MD USA; 4 Department of Health Policy and Management, Bloomberg School of Public Health, Johns Hopkins University, Baltimore, MD USA; 5 Program on Reproductive Health and the Environment, Department of OB/GYN & RS, University of California, San Francisco, CA USA; 6 Department of Environmental and Occupational Health, Dornsife School of Public Health, Drexel University, Philadelphia, PA USA; 7 Urban Health Collaborative, Dornsife School of Public Health, Drexel University, Philadelphia, PA USA; 8 Department of Epidemiology, University of Iowa College of Public Health, 145N. Riverside Drive, Iowa City, IA USA; 9 Center for Emerging Infectious Diseases, University of Iowa College of Public Health, Coralville, IA USA; 10 Department of Biostatistics, Environmental Health Sciences and Epidemiology, College of Public Health, Kent State University, Kent, OH USA; 11 Department of Environmental Health Sciences, University at Albany School of Public Health, State University of New York, Rensselaer, NY USA; 12 School of Public Health, University of California, Berkeley, CA USA

## Abstract

In this comment, we summarize several scientific concerns with the recently published systematic review from O’Connor and colleagues that examined the relationship between proximity to animal-feeding operations and health of individuals in nearby communities. The authors utilized a bias tool not designed for environmental health research, erroneously excluded important studies, and incorrectly interpreted others. As a result, the conclusions drawn in the review misrepresent the evidence from the published literature, limiting its value to policymakers, researchers, and the public.

There is increasing recognition of the value of systematic reviews in the context of decision-making. These reviews are a popular tool in evidence-based medicine [1], and potential opportunities for their application in judging environmental health risks are actively discussed [2–5]. When properly conducted, systematic reviews can serve as critical inputs to the policy process and result in choices about interventions that are in the public’s interest and based on the best available science. Conversely, the use of systematic reviews that suffer from critical design flaws or lack rigorous evaluation of the evidence may hurt public health. We have concern that the recently published systematic review by O’Connor and colleagues [6] on the associations between living near an animal-feeding operation (AFO) and human health falls into the latter category. Although the review integrates some best practice guidelines for conducting systematic reviews, such as developing and registering a protocol beforehand in PROSPERO, the review appears substantially flawed in its approach and conduct; the authors utilized a bias tool that is inappropriate for environmental health research, erroneously excluded important studies, and incorrectly interpreted others. As a result, the ultimate conclusions of the review misrepresent evidence from the published literature. Given that the authors describe their review as having utility for decision-making and prioritizing research, a formal critique of the work is warranted to help calibrate its value for policymakers, researchers, and the public.

The systematic review suffers from shortcomings related to the application of the ROBINS-I (Risk of Bias in Non-randomized Studies of Interventions) tool. The tool was originally developed by the Cochrane Collaboration to evaluate the internal validity of studies of potentially beneficial medical interventions [7]. Direct application to environmental health research is inappropriate because the evidence base and decision-making context differs substantially from clinical settings. For instance, many of the signaling questions to evaluate the study’s internal validity that inquire about intervention-specific details (adherence to the assigned intervention regimen, post-intervention variables influencing study participation, etc.) are not directly relevant to studies of potentially harmful environmental exposures. The use of the ROBINS-I tool in this context will inherently bias conclusions by finding that all included studies are of low quality, given the unavoidable limitations even in well-designed observational epidemiology studies.

Since 2009, there have been ongoing efforts to translate systematic review methods from the clinical sciences to the environmental health decision context, including the development of risk of bias tools in academic and government settings. There are now at least two published methods [2, 8] and several case studies [3, 5, 9–13] that illustrate approaches more directly applicable to review questions in the environmental health decision-making context than ROBINS-I.

The article-screening process and the application of the exclusion criteria were flawed in design and conduct. In the Level 2 screening, the authors specified that articles that did not include “more than one unit of measurement of exposure” would not be considered further in the review. There is no clear definition of this exclusion criterion offered in the text or justification for why the authors chose to exclude such studies. This criterion was used to eliminate eight studies identified in the Level 1 screening from further consideration. Inclusion of these articles would have increased the number of included studies by 50%, indicating that the impact of such an unjustified exclusion may be so strong in this case as to have led to spurious conclusions.

Beyond design flaws, we noted that numerous key papers examining infectious disease and respiratory outcomes related to community exposures to AFOs [14–18] were identified during the Level 1 screening but inappropriately excluded during the Level 2 screening. The rationale presented for excluding these papers was reported in the supplemental material as “the unit of analysis was not at the individual human level or the study looked at occupational exposure only”; however, neither of these statements applied to any of these studies. For example, Casey et al.’s analyses drew on individual-level longitudinal electronic health record data from primary care patients of the Geisinger Health System in Pennsylvania [15]. This study was the first in the USA to demonstrate an association between residential proximity to swine AFOs and manure-applied crop fields and methicillin-resistant *Staphylococcus aureus* (MRSA) infection in a general population sample. The inappropriate exclusion of studies highlights serious concerns related to the rigor and consistency of analysis at the second level of screening and calls into question the validity of conclusions drawn by the authors on the basis of their review. In addition, we note that at least one of our papers [19] that appears relevant to the primary research question was not identified during the Level 1 screening process. Problems with the search strategy may suggest that other key publications could have also been missed, further limiting confidence in the authors’ conclusions.

The authors eliminated ecological studies from their review a priori. A better option may have been to include them while acknowledging the weaknesses and strengths; while inferences about individual-level effects of exposures cannot be inferred from ecologic analyses, these studies have utility in that they establish preliminary evidence of potential associations between exposure and health outcomes, often over large geographies. The authors also limited the scope of their review to the health effects in the community rather than including occupational exposures, despite the notion that workers often reside in communities with their families. While we do not take issue with the exclusion of occupational studies from the systematic review, we note that the environmental health literature offers many examples (e.g., lead, chromium, beryllium) of instances where early warnings arose from higher-level exposures seen in occupational settings [20]. In the case of AFOs, a growing literature exists documenting relationships between occupational exposures and infectious disease and respiratory outcomes [21–27].

Multiple errors in study interpretation and presentation were also noted. For example, the authors stated that Feingold et al. [28] reported an association between MRSA carriage and livestock density, but the Feingold study utilized a case-case analysis comparing MRSA ST-398 to other MRSA strains, not to a control population that would allow such a conclusion to be drawn. Other examples include the erroneous presentation of data from the Avery et al. [29] study, which O’Connor et al. mistakenly list in Table 1 as part of the Community Health Effects of Industrial Hog Operations (CHEIHO) study, rather than as part of a smaller pilot investigation. Also contrary to its presentation in Table 1, Schinasi et al. [30] employed multivariate fixed effects regression models as the primary analytic method.

O’Connor et al.’s evaluation of risk of bias across studies may reflect a misunderstanding of the design and analysis used in some of the papers reported. The authors state, “[b]ecause the studies were cross-sectional in nature, adjustment for confounders would not provide protection against residual confounding.” We disagree with these assertions for two reasons. First, methods exist to evaluate the degree of bias introduced from unmeasured confounders [31] and this residual confounding does not always prevent causal inference from cross-sectional studies [32]. Second, multiple studies included in the review were longitudinal in nature [30, 33, 34]. For example, the CHEIHO study used a case-crossover design and adjusted for within-person, time-invariant confounders. As a result, the models quantified the impact of exposure variability on response variability *within* each person and, in so doing, adjusted for all stable, potentially confounding covariates that varied between people [35]. While it is true that unmeasured time-varying confounding may remain, these models accounted for much of the confounding that is normally a concern [36].

In their discussion section, O’Connor and colleagues call into question studies that are older than 10 years (e.g., [30, 33, 34, 37, 38]). The authors suggest that “dramatic” changes in environmental regulation, animal housing, and manure management may have occurred that would invalidate these studies. However, no evidence supporting this point is presented. Additionally, there is no reason to believe that biologic effects of exposures related to AFOs would change as a function of time.

Policymakers depend on peer-reviewed scientific research as a credible source of information upon which decisions can be built. As a recent example, some of the studies of community risks of AFOs that were excluded in this review [14, 15] and other related research syntheses [39] played an important role in advocacy efforts (e.g., [40, 41]) around two state policies aimed at eliminating non-therapeutic antibiotic use in animal agriculture [42, 43]. Despite the utility of these studies, keeping up with and interpreting the scientific literature is beyond the capacity of most policymakers; thus, the conclusions of systematic reviews can be instrumental in supporting the development of evidence-based policy. When design flaws and less-than-rigorous conduct influence their results, however, the findings of these reviews may be used to adversely impact the policy process or resultant interventions. In contrast to the findings of the authors of this review, we believe there is a considerable and growing body of rigorously conducted scientific evidence that suggests connections between living near AFOs and adverse health outcomes [44]. With a different tool to evaluate the evidence, and a more rigorous application of that tool, it is entirely possible the authors would have come to a different conclusion regarding the community health risks of AFOs.
